# Relative comparison of chronic kidney disease-mineral and bone disorder rat models

**DOI:** 10.3389/fphys.2023.1083725

**Published:** 2023-02-03

**Authors:** Xiaoqiong Zhang, Ting Li, Lijuan Wang, Yanhui Li, Taoren Ruan, Xiaohong Guo, Qin Wang, Xianli Meng

**Affiliations:** ^1^ School of Pharmacy, Chengdu University of Traditional Chinese Medicine, Chengdu, China; ^2^ Department of Pharmacy, Chongqing Hospital of Traditional Chinese Medicine, The Fourth Affiliated Clinical Medical College of Chengdu University of Traditional Chinese Medicine, Chongqing, China; ^3^ School of Pharmacy, Chongqing University of Medical Sciences, Chongqing, China; ^4^ Department of Pathology, Chongqing Hospital of Traditional Chinese Medicine, The Fourth Affiliated Clinical Medical College of Chengdu University of Traditional, Chongqing, China; ^5^ Chongqing Key Laboratory of Traditional Chinese Medicine to Prevent and Treat Autoimmune Diseases, Chongqing Hospital of Traditional Chinese Medicine, The Fourth Affiliated Clinical Medical College of Chengdu University of Traditional Chinese Medicine, Chongqing, China; ^6^ Innovative Institute of Chinese Medicine and Pharmacy, Chengdu University of Traditional Chinese Medicine, Chengdu, China

**Keywords:** CKD–MBD, nephrectomy, Adriamycin nephropathy, unilateral ureteral obstruction, model

## Abstract

**Objective:** The aim of this study is to establish a suitable animal model of chronic kidney disease–mineral and bone disorder (CKD–MBD) by comparing CKD–MBD rat models induced by 5/6 Nx, AN, and UUO, accompanied by a low-calcium and high-phosphorus diet.

**Methods:** Sprague‒Dawley rats were randomly divided into four groups: control group, 5/6 nephrectomy (5/6 Nx) group, Adriamycin nephropathy (AN) group, and unilateral ureteral obstruction (UUO) group. Serum biochemical indices were measured to evaluate renal function, mineral and bone metabolism, the severity of CKD–MBD, and the status of bone transformation. Hematoxylin–eosin staining (HE) and Masson’s trichrome (Masson) staining were used for histopathological analysis of the kidney. Goldner’s trichrome (Goldner) and tartrate-resistant acid phosphatase (TRAP) staining were utilized to observe bone mineralization and osteoclasts in the femur, respectively. Micro-CT images were applied to study the structure of the femur. The expression levels of osterix and cathepsin K in the femur were measured by immunohistochemistry (IHC) to confirm the status of bone transformation.

**Results:** The levels of serum creatinine (Scr) and blood urea nitrogen (BUN) in the 5/6 Nx and AN group rats were significantly higher than those in the control rats, and this change was accompanied by marked changes in the levels of calcium (Ca), phosphate (Pi), intact parathyroid hormone (i-PTH), fibroblast growth factor 23 (FGF23), osteocalcin (OC), and cross-linked C-telopeptide of type 1 collagen (CTX-1); UUO group rats exhibited slight and inconsistent variations in the levels of Scr, BUN, Ca, Pi, i-PTH, FGF23, OC, and CTX-1 in serum. Histopathological analysis of the kidney showed that the UUO group rats suffered serious fibrosis and 5/6 Nx group rats exhibited severe focal calcification. Histopathological analysis of the femur showed that the AN group rats had minimal bone mineralization and that the 5/6 Nx group rats had overactive osteoclasts. Micro-CT revealed that the AN model had the most severe bone destruction and that the 5/6 Nx model had the least severe bone loss among the three models. The expression of cathepsin K in the femur was significantly increased in all models, while the expression of osterix in the femur was only significantly increased in the 5/6 Nx model.

**Conclusion:** 5/6 Nx, AN, and UUO accompanied by a low-calcium and high-phosphorus diet successfully induced CKD–MBD in rats. The 5/6 N_X_ model presented the progression of high-turnover bone disease, with consistency between biochemical indices in serum and histomorphometric analysis of the femur, and the AN and UUO models developed a severe deterioration in bone quantity and severe bone resorption; however, the changes in biochemical indices were subtle in the UUO model, and liver injury was obvious in the AN model.

## 1 Introduction

Chronic kidney disease–mineral and bone disorder (CKD–MBD) is a common complication of chronic kidney disease (CKD), with an incidence in developing countries from 33.3% to 81% of CKD patients (Seyedzadeh et al., 2022). CKD–MBD is accompanied by systemic dysfunction of mineral and bone metabolism, such as abnormalities in serum calcium (Ca), phosphate (Pi), parathyroid hormone (PTH), vitamin D, and fibroblast growth factor 23 (FGF23) metabolism, as well as in bone turnover, mineralization, and volume (TMV). CKD–MBD usually induces skeletal growth defects, fragility, and calcification resulting from extravasation, particularly vascular calcification (Elias et al., 2018; Reiss et al., 2018). Because CKD–MBD contributes to cardiovascular disease, which is the main cause of death in patients with CKD (Reiss et al., 2018; Tani et al., 2020), it is necessary to prevent and treat CKD–MBD as early as possible. However, the present treatments aiming to control serum Ca, Pi, and PTH levels are not effective, and the mortality rate among patients with CKD remains extremely high (Elias et al., 2018). Therefore, an appropriate animal model of CKD–MBD is urgently needed to explore effective strategies to prevent and treat CKD–MBD.

In recent years, 5/6 nephrectomy (5/6 Nx) (Shalhoub et al., 2012; Watanabe et al., 2018; Liu et al., 2021a), adenine with high-phosphorus diet consumption (Tani et al., 2020; Tsuboi et al., 2020), unilateral ureteral obstruction (UUO) (Nordholm et al., 2018; Jiang et al., 2021; Jin et al., 2022), and Adriamycin nephropathy (AN) (Zhang et al., 2019; Li et al., 2020) have been utilized to model CKD–MBD. However, there is a lack of systematic studies comparing these models. To identify a model that accurately mimics the characteristics of CKD–MBD observed in the clinic, we need to determine the differences in CKD–MBD-related manifestations among the models. Therefore, we induced CKD–MBD in rats fed with a low-calcium and high-phosphorus diet (0.6% Ca and 1.2% Pi) by 5/6 Nx, AN, and UUO and then assessed the renal function by measuring serum creatinine (Scr) and blood urea nitrogen (BUN) levels; determined the bone metabolism index by measuring Ca, Pi, and FGF23 content; monitored the levels of key markers associated with bone metabolism, such as intact parathyroid hormone (i-PTH), FGF23, osteocalcin (OC), and cross-linked C-telopeptide of type 1 collagen (CTX-1) in serum; examined histopathological changes in the kidney and femur by hematoxylin–eosin (HE), Masson’s trichrome (Masson), Goldner’s trichrome (Goldner), and tartrate-resistant acid phosphatase (TRAP) staining; performed micro-CT imaging of the femur; and evaluated the expression of osterix and cathepsin K by immunohistochemical (IHC) staining. This systematic study may offer some useful information for choosing an experimental animal model of CKD–MBD.

## 2 Materials and methods

### 2.1 Animals

Sprague‒Dawley rats (male, 6 weeks old, 180 ± 20 g) were purchased from Hunan Slake Jingda Experimental Animal Co., Ltd., (SCXK (Xiang) 2019-0004) and housed under standard conditions in a light-, temperature-, and humidity-controlled environment at the Experimental Animal Center of Chongqing Hospital of Traditional Chinese Medicine (SYXK (Yu) 2020-0001). The rats were provided rodent chow and water *ad libitum*. This study was approved by the Experimental Animal Welfare and the Ethics Committee of Chongqing Hospital of Traditional Chinese Medicine (2022-DWSY-WQ). The experiment complied with the State and hospital’s regulations and ethical requirements for experimental animal studies.

### 2.2 Experimental design

**FIGURE 1:**
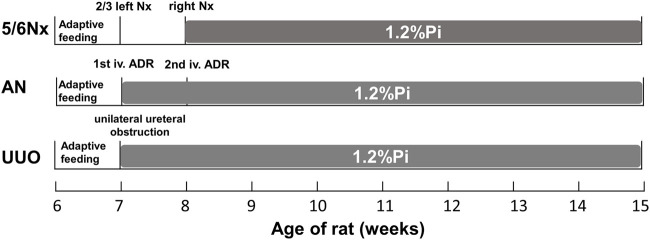
Experimental timeline for establishing the three CKD–MBD models.

Twenty-four rats were divided randomly into the control group, 5/6 N_X_ group, AN group, and UUO group (Figure 1). The control group was fed standard rodent chow but did not undergo experimental operation, and the other groups underwent experimental operation described as follows.

In the 5/6 N_X_ group (Watanabe et al., 2018; Liu et al., 2021a), six rats were anesthetized with isoflurane (Shenzhen RWD Life Science and Technology Co., Ltd., Shenzhen, China) by an R550I small animal anesthesia machine (Shenzhen RWD Life Science and Technology Co., Ltd., Shenzhen, China) and placed on a heating pad to maintain their body temperature. Then, the left flank of each rat was shaved and disinfected with 0.5% iodophor. A 1–1.5 cm incision was made parallel to the ribs to expose the kidney. Then, the renal capsule was stripped, and 1/3 of the upper and 1/3 of the lower poles of the left kidney were removed. The abdominal cavity was closed after the bleeding was stopped by gel foam pads. Seven days later, the right side was prepared in the same way, the renal vessels close to the kidney were ligated, the kidney was removed, and the abdominal cavity was closed. After the second operation, the rats were given a low-calcium and high-phosphorus diet (0.6% Ca and 1.2% Pi) for 7 weeks.

In the AN group (Zhang et al., 2019; Li et al., 2020), six rats were injected with Adriamycin (Shenzhen Wanle Pharmaceutical Co., Ltd., Shenzhen, China) at a dose of 5 mg/kg. One week later, the rats were injected with Adriamycin at a dose of 2 mg/kg. After the first injection, they were fed a low-calcium and high-phosphorus diet (0.6% Ca and 1.2% Pi) for 8 weeks.

In the UUO group (Jiang et al., 2021; Jin et al., 2022), six rats were anesthetized with isoflurane as previously described. Then, the left kidney and ureter were exposed *via* a flank incision, and the left ureter was ligated with 3–0 silk sutures at two points before closing the incision. After the operation, they were fed a low-calcium and high-phosphorus diet (0.6% Ca and 1.2% Pi) for 8 weeks.

### 2.3 Body weight monitoring and specimen collection

The body weight of the rats was monitored every week during the experimental period. At the fifth week after the first operation, the rats were anesthetized with isoflurane, and then blood was taken from the fundus venous plexus’ individual capillaries. At the eighth week after the first operation, the rats were euthanized under isoflurane anesthesia, and blood was collected from the abdominal aorta and centrifuged at 800 × g for 10 min. Serum was collected and kept at −80°C for analysis with commercial kits and enzyme-linked immunosorbent assay (ELISA). The left kidney and both hind femoral bones (2 cm above and below the knee joints) were harvested and stored for histopathological examination and IHC staining.

### 2.4 Biochemical analysis

Scr, BUN, Ca, and Pi levels in serum were measured using commercial kits (Elabscience Biotechnology Co., Ltd., Wuhan, China) and a microplate reader (BioTek Instrument Co., Ltd., Winooski, VT, United States).

### 2.5 Enzyme-linked immunosorbent assay (ELISA)

FGF23, i-PTH, OC, and CTX-1 levels in serum were determined by ELISA kits (Elabscience Biotechnology Co., Ltd., Wuhan, China). All experimental procedures were performed following the manufacturer’s instructions.

### 2.6 Histopathological assessments

Kidney samples collected from the rats under anesthesia were fixed in 4% phosphate-buffered paraformaldehyde (Biosharp Life Sciences, Anhui, China) (pH 7.4) for 24 h and embedded in paraffin. Serial sections (5 µm thick) were obtained, immersed in xylene and alcohol, stained with hematoxylin for 5 min, immersed in 1% acid ethanol (1% HCl in 70% ethanol), rinsed with distilled water, stained with eosin for 3 min, and immersed in alcohol and xylene. Renal fibrosis was assessed by Masson’s trichrome staining according to the manufacturer’s instructions (Servicebio Biological Co., Ltd., Wuhan, China). The sections were mounted using synthetic resin and pathologically examined under a light microscope (Nikon DS-U3, Nikon Corporation, Tokyo, Japan) by a pathologist who was blinded to the treatment groups.

The right femur samples fixed in 4% paraformaldehyde were decalcified with EDTA decalcification agent (Servicebio Biological Co., Ltd., Wuhan, China) for 3 weeks. The specimens were dehydrated, defatted, and sliced with a Leica RM2016 microtome (Leica Microsystems, Shanghai, China) at a thickness of 4 μm, and then HE, Goldner’s, and TRAP staining was performed according to the manufacturer’s instructions (Servicebio Biological Co., Ltd., Wuhan, China).

### 2.7 Micro-CT scanning and 3D reconstruction

The left femur samples fixed with 4% paraformaldehyde were scanned with a micro-CT SkyScan 1276 system (Bruker Analytical Instruments, Kontich, Belgium). The scan settings were as follows: voxel size: 10.157810 μm; medium resolution, 85 kV, 200 uA; 1-mm Al filter; and integration time: 384 ms. Density measurements were calibrated to the manufacturer’s calcium hydroxyapatite (CaHA) phantom. Analysis was conducted with the manufacturer’s evaluation software. Reconstruction was accomplished by NRecon (version 1.7.4.2). 3D images were obtained from contoured 2D images by methods based on a distance transformation of the grayscale original images (CTvox; version 3.3.0). 3D and 2D analyses were performed using CT Analyzer software (version 1.18.8.0). The bone microarchitecture of the distal femur and the region of interest (ROI) was analyzed by determining parameters including the bone mineral density (BMD), total tissue volume (TV), bone volume (BV), bone volume ratio (BV/TV), bone area (BS), bone surface density (BS/TV), trabecular bone number (Tb.N), trabecular bone thickness (Tb.Th), and trabecular bone separation (Tb.Sp).

### 2.8 IHC analysis of femurs

Femur sections were subjected to IHC staining and analyzed as previously described (Liu et al., 2021b). The femur sections were dewaxed and repaired with pepsin antigen retrieval solution (Servicebio Biological Co., Ltd., Wuhan, China). After blocking in bovine serum albumin (BSA) (Servicebio) for 30 min, the sections were incubated with primary antibodies against osterix (Servicebio, 1: 300 dilution) or cathepsin K (Servicebio, 1: 500 dilution) overnight at 4°C. The next day, after being washed three times, the sections were incubated with secondary antibody (Servicebio, 1: 200 dilution) at room temperature in the dark for 50 min. DAB (Servicebio) staining was performed, and tap water was used to stop the reaction. Then, the nuclei were counterstained with hematoxylin (Servicebio) for 3 min. Finally, images were obtained using a microscope (Nikon DS-U3, Nikon Corporation, Tokyo, Japan). The images were analyzed using Image-Pro Plus 6.0 (Media Cybernetics, Inc., Rockville, MD, United States).

### 2.9 Statistical analysis

All data were analyzed using GraphPad Prism version 8.0 (GraphPad Software Inc., San Diego, CA, United States). Comparisons between groups were performed using one-way ANOVA. The data are presented as the mean ± standard deviation (SD). Statistical significance was set at *p* < 0.05.

## 3 Results

### 3.1 Body weight gain occurred more slowly in all three CKD–MBD models

**FIGURE 2:**
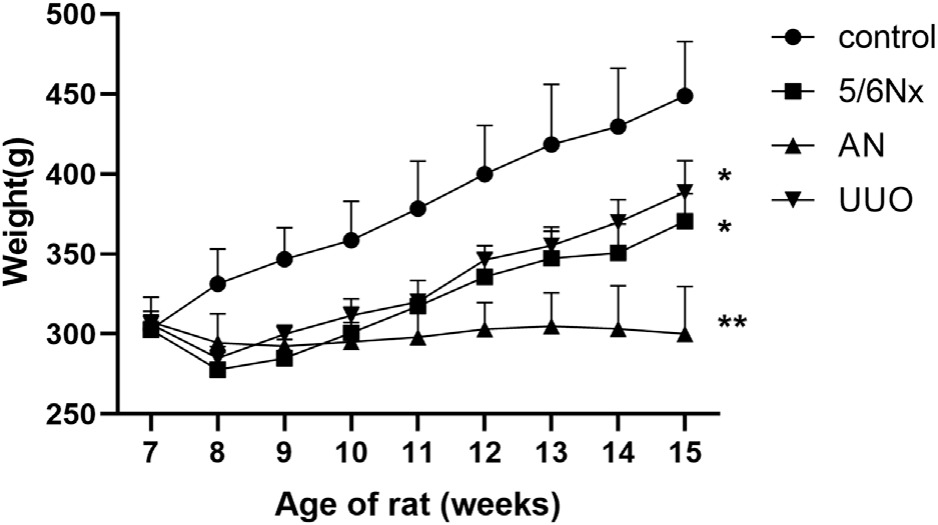
Curves of body weight changes in the different model groups. *p < 0.05 and **p < 0.01 vs. the control group. Data are presented as mean ± SD. *n* = 6.

Compared with those in the control group, the rats in all three CKD–MBD model groups showed slower body weight gain. Notably, the body weight of the AN group rats did not show any increase over the 8 weeks but instead decreased. Statistical analysis revealed that the body weights of rats in the 5/6 Nx group, the UUO group, and the AN group were significantly lower than those of rats in the control group at the age of 15 weeks (Figure 2).

### 3.2 The degree of renal damage was different among the three CKD–MBD models

**FIGURE 3:**
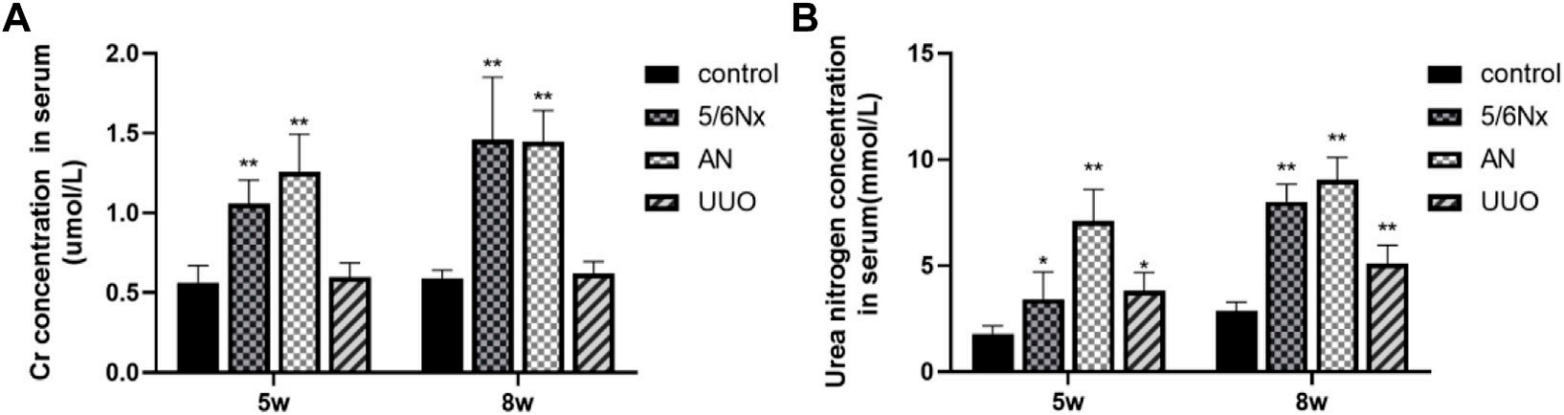
Levels of Scr and BUN in serum in different models. **(A)** Levels of Scr in serum at the fifth week and eighth week. **(B)** Levels of BUN in serum at the fifth week and eighth week. *p < 0.05 and **p < 0.01 vs. the control group. Data are presented as mean ± SD. *n* = 6.

The renal function of the CKD–MBD model rats was evaluated by measuring Scr and BUN levels in serum at the fifth and eighth weeks. Compared with those in the control group, the levels of Scr and BUN in the 5/6 N_X_ group and the AN group were significantly increased (Figure 3). Although the BUN level was increased in the UUO group compared to the control group (Figure 3B), the Scr level showed no difference between the two groups (Figure 3A). The results indicated that renal function in the 5/6 N_X_ and AN groups was severely damaged; however, the rats in the UUO group showed no obvious damage.

### 3.3 Ca and Pi levels in serum were altered in the three CKD–MBD models

**FIGURE 4:**
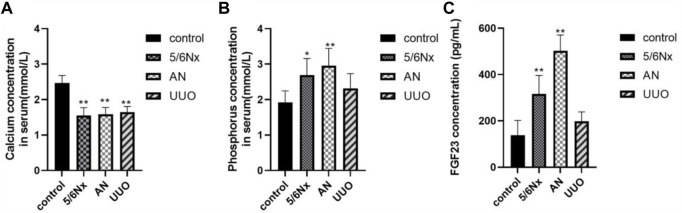
Levels of Ca, Pi, and FGF23 in serum in different models. **(A)** Levels of Ca in serum at the eighth week. **(B)** Levels of Pi in serum at the eighth week. **(C)** Levels of FGF23 in serum at the eighth week. *p < 0.05 and **p < 0.01 vs. the control group. Data are presented as mean ± SD. *n* = 6.

Ca is very important for health, and severe hypocalcemia can cause convulsions, arrhythmia, and even sudden death (Moe, 2017). Excessive retention of Pi in the body can cause a wide range of conditions, such as vascular calcification, hyperparathyroidism, hypocalcemia, impaired bone mineralization, dysregulated cell signaling, and cell death (Rastogi et al., 2021; Sprague et al., 2021). The levels of Ca and Pi in serum were determined to evaluate mineral and bone metabolism. FGF23, which is secreted primarily by osteoblasts and osteocytes, is a physiological regulator of circulating phosphate and vitamin D levels, and it has been reported that the serum level of FGF23 is increased with an increased dietary phosphate load (Vogt et al., 2019; Latic and Erben, 2022). It is also commonly believed that CKD–MBD is associated with the elevation of serum i-PTH and FGF23 levels (Cannata-Andía et al., 2021). Therefore, the serum levels of FGF23 were also measured to confirm disturbances in mineral and bone metabolism in the three CKD–MBD models. At 5 weeks after 5/6 Nx, AN, or UUO, no significant changes were observed in Ca and Pi levels (*p* > 0.05), except for the Pi level in the AN group (Supplementary Figures S1A, B). However, at 8 weeks after modeling, all the rats in the three CKD–MBD model groups exhibited a significant decrease in Ca levels and an increase in Pi levels (*p* < 0.05, except for the Pi level in the UUO group) (Figures 4A, B). In accordance with the variation in the levels of Pi, the levels of FGF23 were markedly increased in the 5/6 Nx and AN model rats (*p* < 0.05) (Figure 4C). These results suggest that the 5/6 Nx and AN models exhibit disturbances in mineral and bone metabolism accompanied by elevation of serum Ca, Pi, and FGF23 levels. However, in the UUO model rats, only the Ca level was significantly lower than that in the control group (*p* < 0.05) (Figure 4A).

### 3.4 Bone turnover was disturbed in the three CKD–MBD models

**FIGURE 5:**
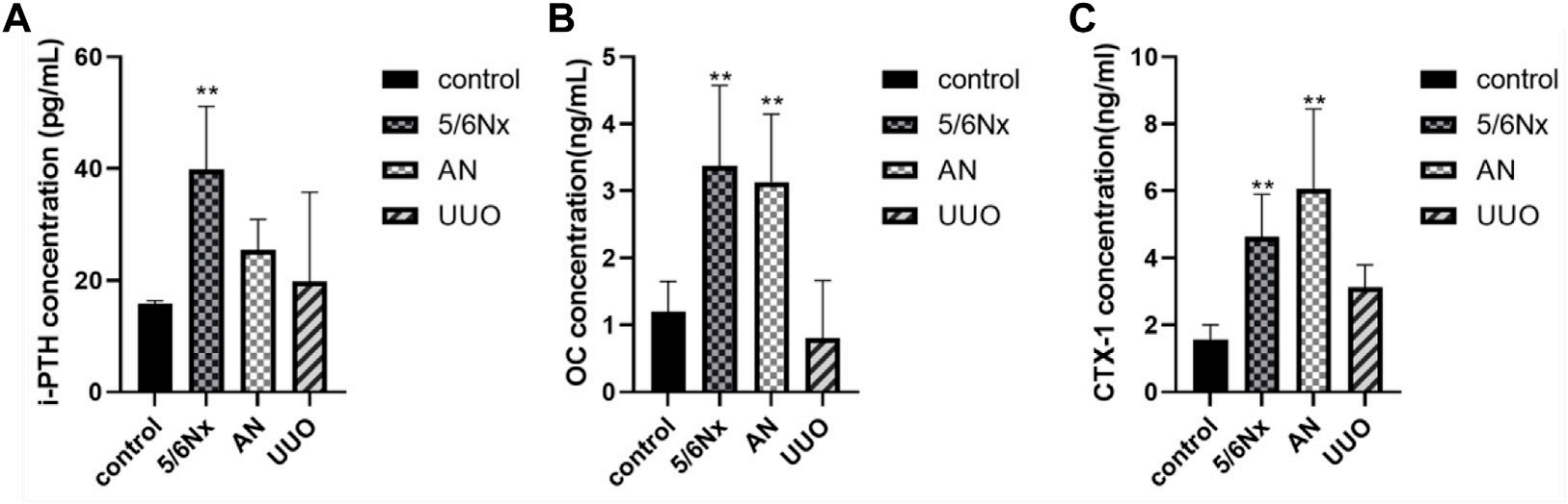
Levels of i-PTH, OC, and CTX-1 in serum in different models. **(A)** Levels of i-PTH in serum in different models. **(B)** Levels of OC in serum in different models. **(C)** Levels of CTX-1 in serum in different models. *p < 0.05 and **p < 0.01 vs. the control group. Data are presented as mean ± SD. *n* = 6.

According to the CKD–MBD guidelines (Chiang, 2017; Ketteler et al., 2017; Baptista et al., 2020; GBD Chronic Kidney Disease Collaboration, 2020; D'Arrigo et al., 2022), i-PTH, a polypeptide hormone produced and secreted by parathyroid chief cells, plays an important role in bone metabolism, and plasma calcium and phosphorus concentrations need to be monitored in adult patients at the beginning of stage 3 CKD. The guidelines (Waziri et al., 2019) recommend that the levels of osteocalcin (OC), a marker of bone formation, and the carboxy-terminal cross-linked telopeptide of type 1 collagen (CTX-1), a marker of bone absorption, be measured when possible. Therefore, the levels of i-PTH, OC, and CTX-1 in serum were measured to confirm the status of bone turnover in the models. Eight weeks after modeling, the levels of i-PTH were significantly increased in the 5/6 Nx group compared with the control group (Figure 5A), indicating that the 5/6 Nx model rats suffered severe high-turnover bone disease; consistent with the levels of i-PTH, the levels of OC and CTX-1 in the 5/6 Nx group were higher than those in the control group (Figures 5B, C), also demonstrating high-turnover bone disease. The levels of i-PTH (Figure 5A), OC, and CTX-1 were not significantly different between the UUO group and the control group (Figures 5B, C). In the AN group, the levels of OC and CTX-1 were significantly higher than those in the control group (Figures 5B, C), and the i-PTH level was increased. These data indicate that the 5/6 Nx model and the AN model might suffer high-turnover bone disease, while the UUO model might show low turnover.

### 3.5 Histopathological staining revealed renal injury in all three CKD–MBD models

**FIGURE 6:**
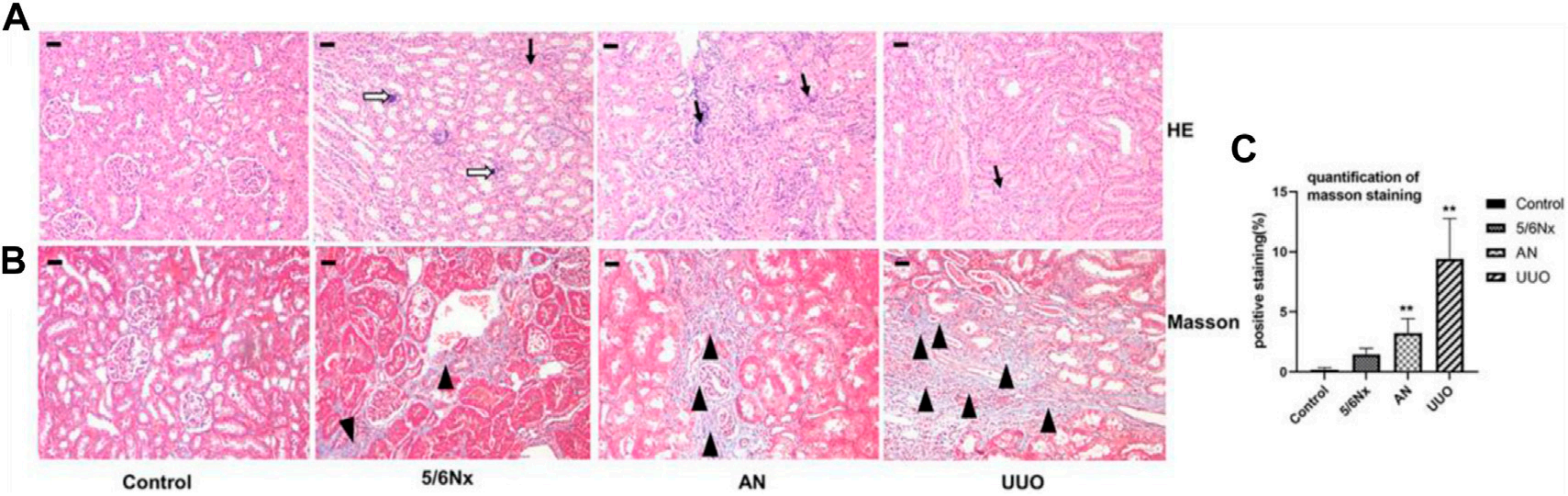
Histopathological analysis of kidneys in different models. **(A)** Representative images of HE staining; × 100, scale bar = 10 μm. Chronic inflammatory cell infiltration: black arrow; focal calcification: white hollow arrow. **(B, C)** Representative images of Masson’s staining for the assessment of fibrosis showing significant differences in the degree of fibrosis (expressed as the percentage of the positively stained area). × 100, Scale bar = 10 μm, renal interstitial fibrosis: black triangle. *N* = 3.

HE staining showed that all the rats in the three model groups exhibited pathological alterations in the kidney, including renal tubular epithelial cell edema and inflammatory cell infiltration (indicated by the black arrow), to a certain degree, but focal calcification (indicated by the white hollow arrow) was observed only in the 5/6 Nx group (Figure 6A). Masson’s staining revealed that renal interstitial fibrosis (indicated by the black triangle) occurred in all model rats, and the most severe fibrosis was observed in the UUO group (Figures 6B, C). All these results revealed that the kidneys were injured in all three CKD–MBD models.

### 3.6 Histopathological alterations in the femur were observed in all three CKD–MBD models

**FIGURE 7:**
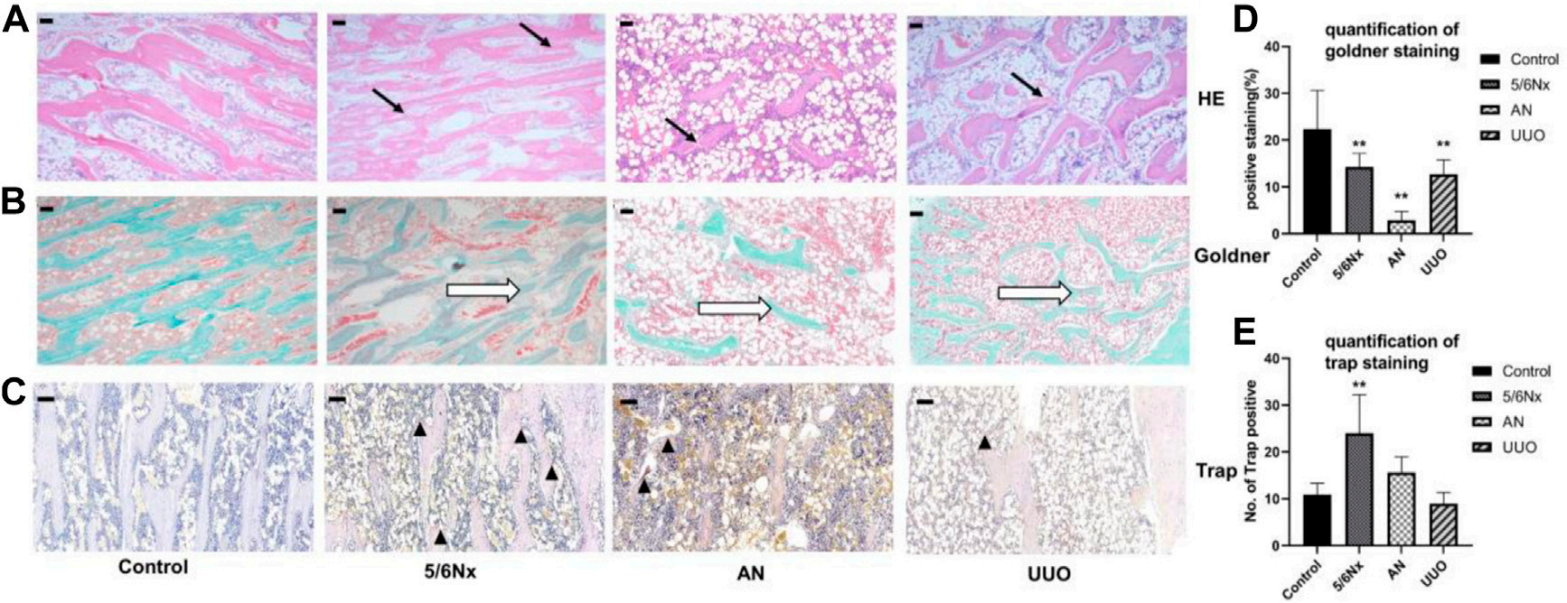
Histopathological analysis of femurs in different models. **(A)** Representative images of HE staining, × 40, scale bar = 200 μm. Trabecular bone: black arrows. **(B, D)** Representative images of Goldner’s staining for assessment of newly formed bone areas (expressed as the percentage of the positively stained area); × 40, scale bar = 200 μm. Bone mineralization: white hollow arrow. **(C, E)** Representative images of TRAP staining for assessment of the number of TRAP-positive cells (expressed as the of TRAP-positive cells). × 100, Scale bar = 10 μm, osteoclasts: black triangle. *N* = 3.

HE staining of femurs showed some degree of trabecular bone disorder (indicated by the black arrows) in all model rats, with the more severe disorder being observed in the AN group (Figure 7A). Goldner’s staining was used to visualize bone mineralization (green). Decreased bone mineralization (indicated by the white hollow arrow) was observed in all three CKD–MBD models. In particular, the reduction in bone mineralization was most severe in the AN group (Figures 7B, D). TRAP staining was used to visualize osteoclasts (pink), and it showed an increased number of osteoclasts (indicated by the black triangle) in all three models, with a particularly remarkable change in the 5/6 Nx model (Figures 7C, E).

### 3.8 Micro-CT 3D imaging showed destruction of femur structure in the three models

**FIGURE 8:**
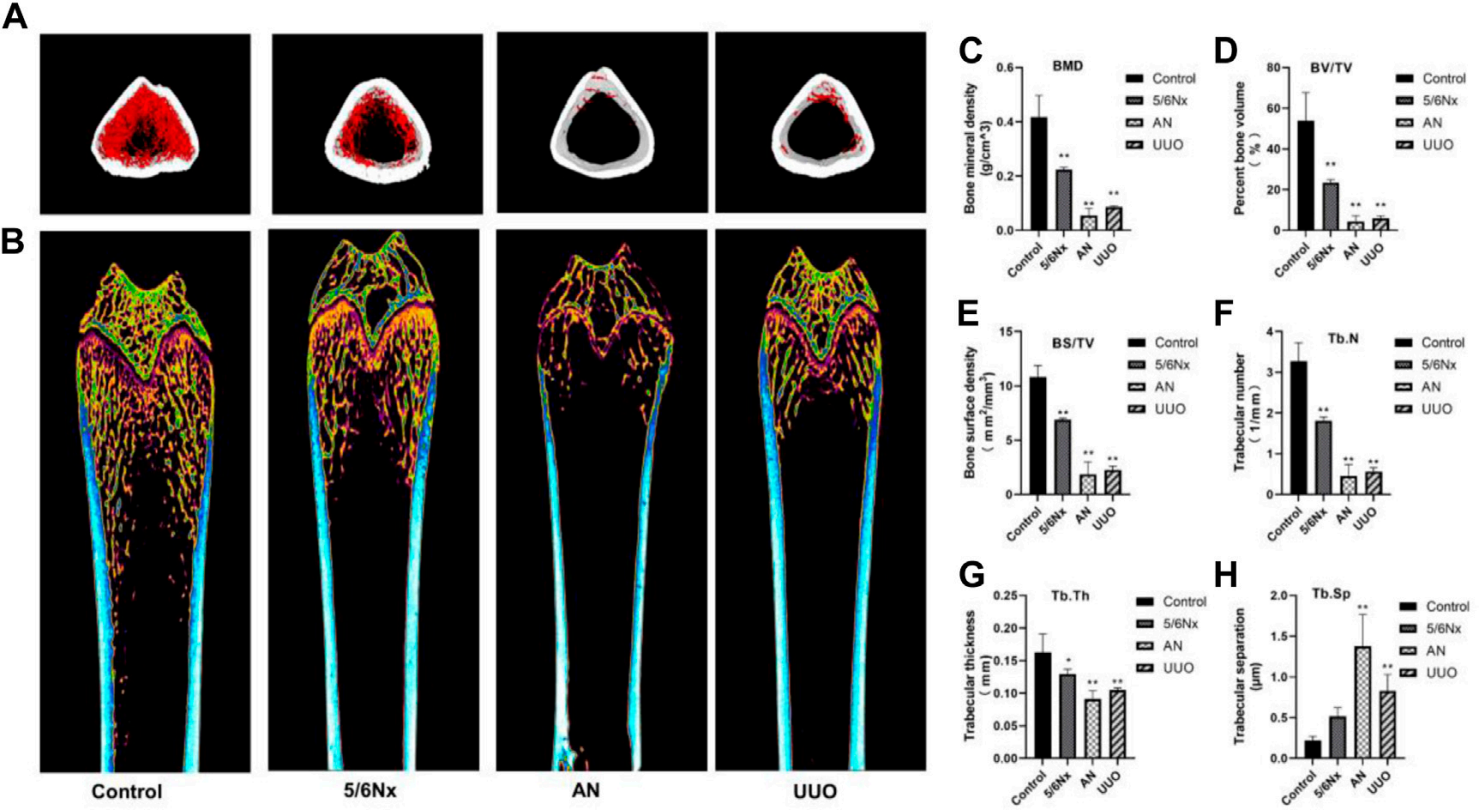
Image of femurs from different models. **(A, B)** Representative micro-CT 3D reconstruction images of femurs from the three model groups. **(C–H)** Parameters of bone histomorphometry. *p < 0.05 and **p < 0.01 vs. the control group. Data are presented as mean ± SD. *n* = 4.

Micro-CT 3D reconstructions of femurs showed that the structure of the distal femur was destroyed in all the three model groups; however, the damage was less severe in the 5/6 Nx group than in the other groups (Figures 8A, B). The BMD of the distal femur in the model groups was significantly lower than that in the control group (Figure 8C). Similar changes were also observed by assessing the BV/TV and BS/TV (Figures 8D, E). Evaluation of morphological parameters of the femur showed that the Tb.N and Tb.Th of the three models were significantly lower than those of the control group (Figures 8F, G), while the Tb.Sp of the AN and UUO models were significantly higher than that of the control group (Figure 8H). The results indicate that the 5/6 Nx group had the least severe degeneration and the AN group had the most severe degeneration.

### 3.9 Osterix and cathepsin K levels in the femur were different among the three models

**FIGURE 9:**
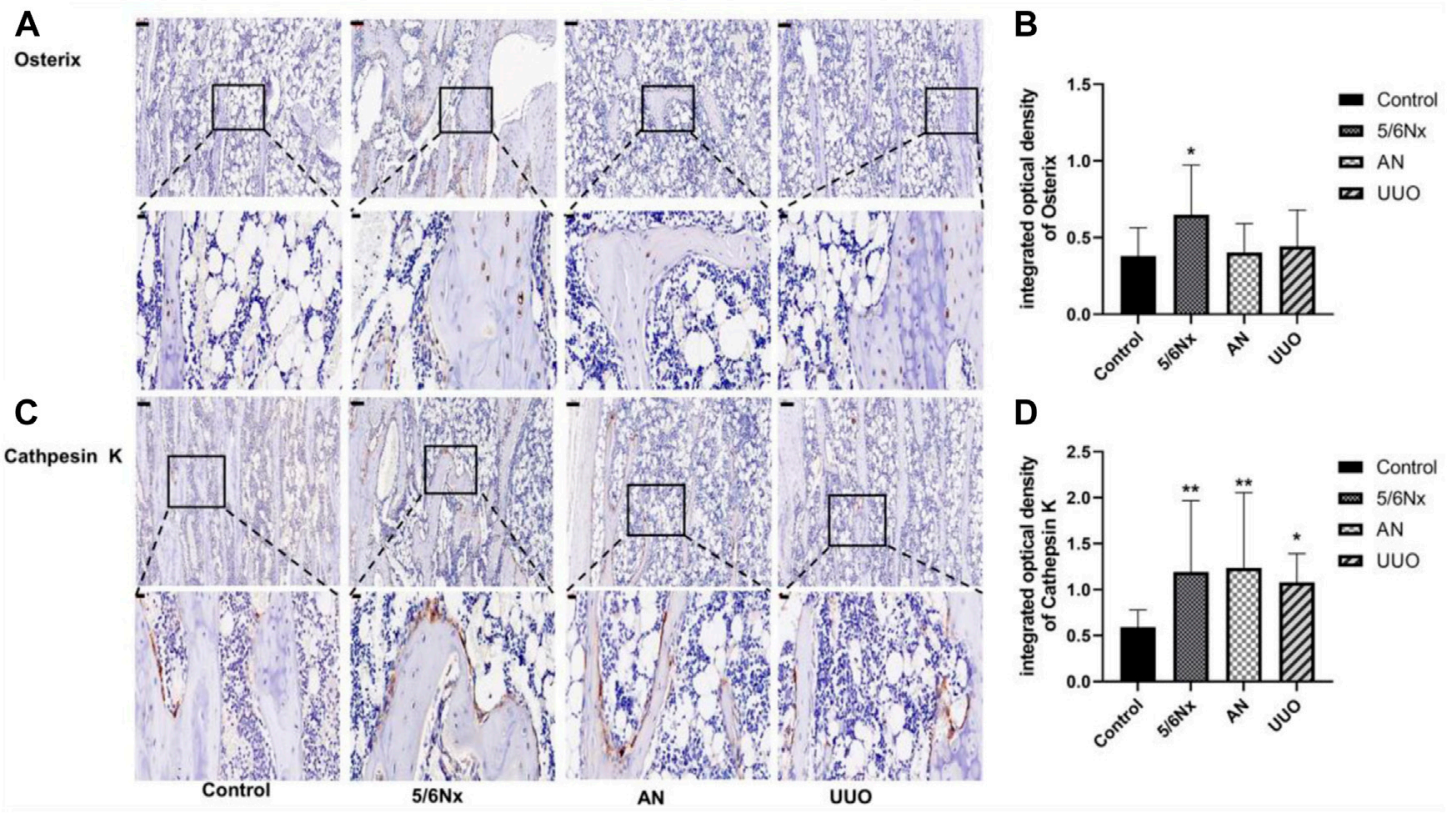
Expression of osterix and cathepsin K in the femur in the three models. **(A)** Protein expression of osterix in the femur; IHC staining, × 10, scale bar = 100 μm; IHC staining, × 40, scale bar = 20 μm. **(B)** Mean density of osterix staining in the femur. **(C)** Protein expression of cathepsin K in the femur; IHC staining, × 10, scale bar = 100 μm; IHC staining, × 40, scale bar = 20 μm. **(D)** Mean density of cathepsin K staining in the femur. Data are presented as the mean ± SD. *n* = 3.

According to the experimental results reported previously, the bone volume in the model rats was changed. Therefore, the levels of osterix, a typical marker of bone formation, and cathepsin K, a marker for bone resorption, were examined to assess bone formation and bone resorption. Although the expression of osterix in all the three model groups was increased, it was only significantly increased in the 5/6 Nx model compared with the control group (Figures 9A, B). Additionally, the expression of cathepsin K in the three model groups was much higher than that in the control group (Figures 9C, D). These results indicate that bone formation and bone resorption were increased in the 5/6 Nx model rats compared with the AN and UUO models.

## 4 Discussion

Patients with CKD experience mineral and bone metabolism disturbance, which results in an increased risk of fracture, especially for those on dialysis (D'Arrigo et al., 2022). It is commonly believed that bone turnover patterns are altered with progression of chronic kidney disease. Increasing evidence indicates that a high prevalence of low bone turnover first occurs in the early stages of CKD, with adynamic bone disease being the predominant form, and that renal function progressively deteriorates. Persistent PTH secretion may lead to secondary hyperparathyroidism, which results in osteitis fibrosa; a high-turnover bone disease resulting mainly from secondary hyperparathyroidism; and finally deterioration in bone quantity with prominent bone resorption in end-stage renal disease (Drüeke and Massy, 2016; Massy and Drueke, 2017; Liu et al., 2018; El-Husseini et al., 2022).

In the present study, we generated rat models of bone disease associated with renal insufficiency by 5/6 nephrectomy, Adriamycin injection, and UUO accompanied by consumption of a low-calcium and high-phosphorus diet to provide information for the study of CKD–MBD. The 5/6 Nx model animals, which were fed a high-phosphorus diet to induce hyperphosphatemia, suffered acute injury with creatinine clearance that was approximately equivalent to late stage 4 or 5 CKD in humans (Wanna-Udom et al., 2022). Therefore, 5/6 Nx model rats develop severe hyperphosphatemia and hyperparathyroidism after acute kidney injury, which promote the progression of CKD–MBD. Because Adriamycin interferes with membrane proteins and podocyte cytoskeletal proteins and can thus impair podocyte function and albuminuria filtration and ultimately lead to glomerular sclerosis and renal failure, the AN model is the classic model of focal segmental glomerulosclerosis (Dou et al., 2020). Moreover, the UUO model is a well-established animal model used to explore tubulointerstitial injury and progressive fibrosis (Wang et al., 2020). Complete ureteral obstruction initiates a sequence of events in the obstructed kidney, including activation of fibroblasts and EMT, leading to a massive increase in the number of myofibroblasts during the early stage, ECM accumulation in tubulo-interstitium, collagen formation, and eventually end-stage obstructive nephropathy after approximately 2 weeks (Humphreys, 2018; Martínez-Salgado et al., 2022). Thus, UUO results in renal fibrosis, especially tubular interstitial fibrosis. Therefore, the different renal damage models generated in this study are beneficial for comprehensively studying bone turnover accompanied by disturbances in mineral and bone metabolism.

In CKD diagnosis, renal function is always evaluated by measuring the levels of Scr and BUN in serum, and then the estimated glomerular filtration rate (eGFR) is calculated based on the level of Scr. Furthermore, the stages of CKD are classified according to the eGFR level (Wan et al., 2022; Zhang et al., 2022). With the progression of CKD, the levels of Scr and BUN in serum increase and the eGFR decreases. As a complication of CKD, CKD–MBD often silently begins in the early stages of CKD and is usually diagnosed in G3b–G4 stages of CKD (Elias et al., 2018; Ketteler et al., 2018). Therefore, CKD–MBD patients always have extremely high levels of Scr and BUN in serum. In this study, renal injury in the different models was evaluated by measuring Scr and BUN levels in serum. The data showed that 5/6 Nx and AN led to severe renal injury at the fifth week after the first operation; furthermore, renal failure progressed within the experimental period, especially in the 5/6 Nx model rats. Comparatively, Scr and BUN levels increased slowly in the UUO group; a reasonable explanation for this is that the normal contralateral kidney has a compensatory effect on renal excretion function, which is consistent with a previous report (Martinez-Klimova et al., 2019). Similarly, the difference in the results of kidney histopathology can be explained when whole or focal damage is taken into full consideration. Thus, the aforementioned results indicate that all the models caused renal injury; the 5/6 Nx model rats exhibited obvious damage, while the UUO model rats exhibited slight damage.

According to the CKD–MBD guidelines (Ketteler et al., 2017; GBD Chronic Kidney Disease Collaboration, 2020), the levels of Ca, Pi, and i-PTH in serum should be monitored in adult patients at the beginning of stage 3 CKD, and then the severity of CKD–MBD should be assessed based on the level of i-PTH. Extremely high levels of i-PTH indicate high-turnover bone disease (Laowalert et al., 2020) and *vice versa*. In this study, accumulation of Pi, exhaustion of Ca, and excessive secretion of i-PTH in serum were observed in the 5/6 Nx model rats at the eighth week of the experiment, and the variation trend was consistent with that observed in CKD–MBD patients; elevation of serum FGF23 levels, which corresponded with increases in Pi and i-PTH levels, was also observed in the 5/6 Nx model rats. Therefore, it is clear that 5/6 Nx induced high-turnover bone disease in rats under our experimental conditions. However, UUO model rats exhibited less marked changes in the levels of Pi, i-PTH, and FGF23 during the experiment. Because both serum i-PTH and FGF23 levels can be used to predict the occurrence of high-turnover bone disease, but not low-turnover bone disease (Massy and Drueke, 2017), it is difficult to distinguish the types of bone turnover in UUO model rats according to the serum levels of Pi, i-PTH, and FGF23. A previous study reported that serum sclerostin is a good predictor of low bone turnover and low bone volume (Massy and Drueke, 2017). Unfortunately, the serum levels of sclerostin do not correlate with changes in bone mineral density in patients with osteoporosis and are of limited diagnostic value in this disease (Figurek et al., 2020; Dincel and Jørgensen, 2022; Vavanikunnel et al., 2022) because sclerostin acts locally in the bone microenvironment, and the serum level of sclerostin does not reflect changes in sclerostin expression by osteocytes (Dreyer et al., 2021). Furthermore, it has been reported that romosozumab, a humanized anti-sclerostin antibody, is associated with an increased incidence of cardiovascular events, limiting the diagnostic application of serum sclerostin. Therefore, serum sclerostin was not used as a monitoring index in the present study, although sclerostin may aggravate PTH resistance in CKD (Canalis, 2018). In the AN model rats, accumulation of Pi, exhaustion of Ca, and excessive secretion of FGF23 in serum were observed at the eighth week of the experiment; however, excessive elevation of i-PTH levels was not observed, making it difficult to distinguish the different forms of bone turnover. However, the levels of OC and CTX-1 in serum were abnormally increased in the 5/6 Nx model rats and the AN models rats, which confirmed that the 5/6 Nx model rats and the AN model rats suffered from high-turnover bone disease. On the other hand, the levels of OC and CTX-1 in the UUO model rats showed no obvious change, so it was difficult to determine the forms of bone turnover induced by UUO according to the serum indices only.

In addition to hyperphosphatemia, hypocalcemia, and secondary hyperparathyroidism, active proliferation of osteoblasts and osteoclasts is typical of high-turnover bone disease and leads to excessive bone formation and resorption and eventually bone destruction because of the lack of Ca and overload of Pi (Baptista et al., 2020; Ferreira et al., 2021; Jorgensen et al., 2022). Therefore, bone destruction is not commonly observed in the early stage of CKD–MBD and progresses in the later stages of CKD–MBD. Bone biopsy is the gold standard for diagnosing CKD–MBD; however, it is not strongly recommended because of the invasiveness of the procedure and the difficulty of clinical implementation (Chiang, 2017; Salam et al., 2021). In this study, histopathological analysis of the femur showed that all the experimental methods caused some degree of bone damage, such as a reduction in the number of trabeculae, a decrease in bone mineralization, and overactivation of osteoclasts. In particular, osteoclasts were overactive in the 5/6 Nx model rats, whereas bone mineralization was decreased and trabeculae were degraded in the AN model rats and UUO model rats, meaning that the 5/6 Nx model rats all had high-turnover bone disease and that the AN model rats and UUO model rats had later-stage CKD–MBD. Micro-CT images and measurement of femur parameters, such as BMD, BV/TV, Tb.Th, and Tb.N, all demonstrated that AN resulted in the most severe femur destruction and that 5/6 Nx caused minimal damage relative to the other two techniques, which is consistent with the histopathological analysis of the femur; these results indicate that the 5/6 Nx model rats experienced high bone turnover accompanied by increased bone formation and resorption and that the AN model rats and UUO model rats had later-stage CKD–MBD and suffered severe bone destruction. The expression of osterix and cathepsin K was assessed by IHC, and the results verified that bone formation and resorption were increased in the 5/6 Nx model rats and that bone resorption was increased in the AN model rats and UUO model rats, which is consistent with the histopathology results and micro-CT images.

In UUO model rats, the serum levels of creatinine, Pi, i-PTH, FGF23, OC, and CTX-1 were similar to those in the control group; however, the histopathological analyses micro-CT imaging and IHC all showed that UUO model rats exhibited increased bone resorption. This inconsistency might be attributed to the compensatory effect of the normal contralateral kidney on renal excretion function, which makes it difficult to determine the metabolic status of bone according to serum biochemical indices. According to a previous report, obstructive nephropathy usually occurs within approximately 2 weeks in end-stage CKD (Humphreys, 2018; Martínez-Salgado et al., 2022). Therefore, it is also possible that we fail to detect an increase in serum creatinine, Pi, i-PTH, FGF23, OC, and CTX-1 levels due to the timing of our blood draws. In brief, the UUO model suffered drastic damage with subtle biochemical changes and should be used carefully in the study of CKD–MBD, and bone histomorphometry should be the primary method for determining the form of bone turnover, with serum biochemical indices being less informative.

AN resulted in rapid progression of renal injury during the early period of the experiment; eventually, obvious bone destruction was observed at the end of this experiment. Unfortunately, we did not observe the transformation of bone formation and absorption in the femur, and it is possible that we did not collect femur samples at the most appropriate time. However, utilizing AN to model bone disease and study the course of progressive CKD in rats and humans may provide insight into both normal and abnormal physiology if the timeline of experiments is selected prudently as AN can rapidly induce severe kidney injury. It is worth noting that we observed focal swelling of hepatocytes, multifocal focal necrosis, inflammatory cell infiltration around the portal area, and fibrous tissue hyperplasia in the AN model rats, suggesting that Adriamycin may lead to liver injury (Supplementary Figure S2); thus, the scope of the application of the model should be evaluated.

5/6 Nx induced remarkable variations in Ca, Pi, and i-PTH levels in serum and progression of renal injury. It also led to high turnover of bone formation and absorption according to the levels of OC and CTX-1. These changes were consistent with the findings of histopathological staining and micro-CT imaging. Thus, the 5/6 Nx model can be used to easily investigate the progression of high-turnover bone disease on this experimental schedule.

Overall, 5/6 Nx, AN, and UUO accompanied by a low-calcium and high-phosphorus diet successfully induced CKD–MBD in rats. The AN and UUO models exhibited deterioration in bone quantity with rapid and severe bone resorption at the end of the experiment; however, the changes in the biochemical indices were subtle in the UUO model, and liver injury was obvious in the AN model. The 5/6 Nx model presented high-turnover bone disease at the end of the experiment, with consistency between serum biochemical indices and femur histomorphometric parameters.

## 5 Conclusion

In conclusion, 5/6 Nx, AN, and UUO accompanied by a low-calcium and high-phosphorus diet can result in CKD–MBD. On our experimental schedule, the 5/6 Nx model exhibited high-turnover bone disease, which is easy to evaluate according to serum biochemical indices and histomorphometry. The AN and UUO models presented severe bone resorption and deterioration in bone quantity at the end of the experiment, according to histomorphometric analysis of the femur.

## Data Availability

The original contributions presented in the study are included in the article/Supplementary Material; further inquiries can be directed to the corresponding authors.
