# Supplementary material for: Relative comparison of chronic kidney disease-mineral and bone disorder rat models
Source: Front Physiol. 2023 Feb 3;14:1083725. doi: 10.3389/fphys.2023.1083725 (PMC9936098; doi:10.3389/fphys.2023.1083725)
Supplement: Supplementary file 1 [file DataSheet1.pdf]

Relative Comparison of Chronic Kidney Disease-Mineral and Bone Disorder Rat Models

SUPPLEMENTARY MATERIAL

Ca and Pi in Serum Were Altered in the Three CKD-MBD Models

At 5 weeks after 5/6N$_X$, AN, and UUO, almost no significant changes were observed in Ca and Pi levels (*P*>0.05), except for the Pi level in the AN group(Figure S1).

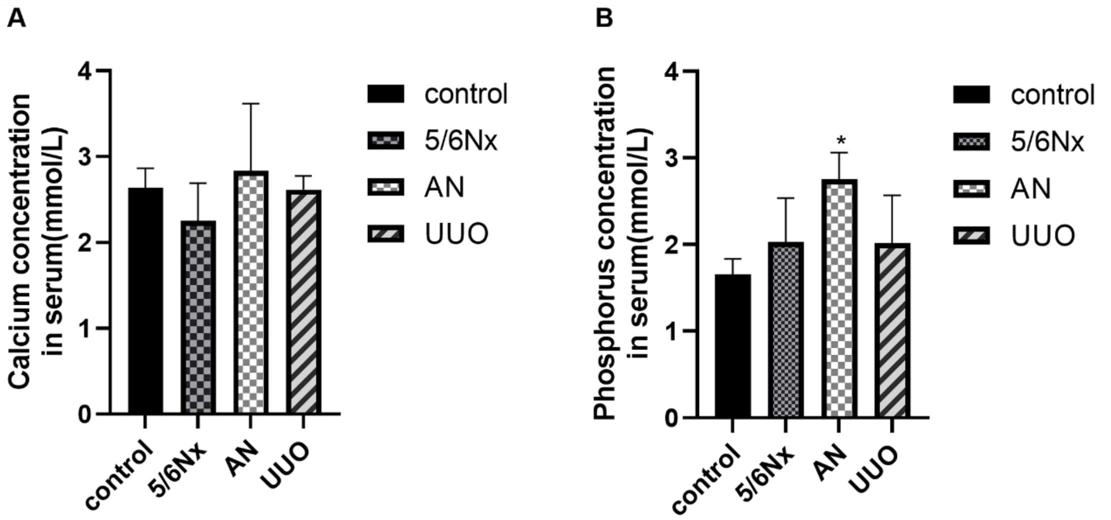

FIGURE S1 | Levels of Ca and Pi in serum in different models at 5 week. **(A)** The levels of Ca in serum at 5th week. **(B)** The levels of Pi in serum at 5th week. * *P*<0.05 and ** *P*<0.01 vs. the control group. Data are presented as mean ± SD. n=6.

Liver injury in the model rats

HE staining of liver tissue displayed focal swelling of hepatocytes, multifocal focal necrosis, inflammatory cell infiltration around the portal area, and fibrous tissue hyperplasia in the AN group, but no pathological changes were observed in the 5/6Nx and UUO models (Figure S2).

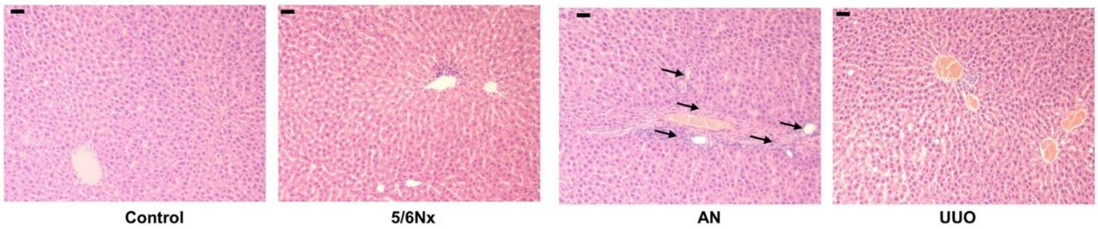

FIGURE S2 | Histopathology of liver in different models. Representative images of HE staining, × 100, Scale bar=10 μm, focal necrosis: black arrow. n=3.
